# New horizon in platelet function: with special reference to a recently-found molecule, CLEC-2

**DOI:** 10.1186/s12959-016-0099-8

**Published:** 2016-10-04

**Authors:** Yukio Ozaki, Shogo Tamura, Katsue Suzuki-Inoue

**Affiliations:** 1 Fuefuki Central Hospital, 47-1 Yokkaichiba, Isawa, Fuefuki, 406-0032 Yamanashi Japan; 2 Department of Laboratory Medicine, University of Yamanashi, 1110 Shimokato, Chuo, Yamanashi 409-3898 Japan; 3 Department of Pathophysiological Laboratory Sciences, Nagoya University Graduate School of Medicine, 1-1-20, Oosachi Minami, Higashi, Nagoya, 461-8673 Aichi Japan

**Keywords:** Platelets, Thrombosis, Beyond hemostasis, Immunity, CLEC-2, Lymphangiogeneis, Smooth muscle cells, Megakaryopoiesis

## Abstract

Platelets play a key role in the pathophysiological processes of hemostasis and thrombus formation. However, platelet functions beyond thrombosis and hemostasis have been increasingly identified in recent years. A large body of evidence now exists which suggests that platelets also play a key role in inflammation, immunity, malignancy, and furthermore in organ development and regeneration, such as the liver.

We have recently identified CLEC-2 on the platelet membrane, which induces intracellular activation signals upon interaction of a snake venom, rhodocytin. Later we discovered that podoplanin, present in renal podocytes and lymphatic endothelial cells, both of which are not accessible to platelets in blood stream, is an endogenous ligand for CLEC-2. In accord with our expectation, platelet-specific CLEC-2 knockout mice have a phenotype of edema, lymphatic vessel dilatation, and the presence of blood cells in lymphatic vessels. It is suggested that lymphatic/blood vessel separation during the developmental stage is governed by cytokines released from platelets activated by the interaction between platelet CLEC-2 and podoplanin present on lymphatic endothelial cells.

Recombinant CLEC-2 bound to early atherosclerotic lesions and normal arterial walls, co-localizing with vascular smooth muscle cells (VSMCs). Flow cytometry and immunocytochemistry showed that recombinant CLEC-2, but not an anti-podoplanin antibody, bound to VSMCs, suggesting that CLEC-2 ligands other than podoplanin are present in VSMCs. Protein arrays and Biacore analysis were used to identify S100A13 as a CLEC-2 ligand in VSMCs. S100A13 was released upon oxidative stress, and expressed in the luminal area of atherosclerotic lesions.

Megakaryopoiesis is promoted through the CLEC-2/podoplanin interaction in the vicinity of arterioles, not sinusoids or lymphatic vessels. There exist podoplanin-expressing bone-marrow (BM) arteriolar stromal cells, tentatively termed as BM fibroblastic reticular cell (FRC)-like cells, and megakaryocyte colonies were co-localized with periarteriolar BM FRC-like cells in the BM. CLEC-2/podoplanin interaction induces BM FRC-like cells to secrete CCL5 to facilitate proplatelet formation. These observations indicate that a reciprocal interaction with between CLEC-2 on megakaryocytes and podoplanin on BM FRC-like cells contributes to the periarteriolar megakaryopoietic microenvironment in mouse BM.

## Background

Platelets play a key role in the pathophysiological processes of hemostasis and thrombus formation. However, platelet functions beyond thrombosis and hemostasis have been increasingly identified in recent years. This review reports on the newly identified platelet functions beyond hemostasis, especially with the reference to CLEC-2.

## Introduction

In primitive organisms such as horseshoes crabs, except for red blood cells, there is only one additional type of blood cells, hemocytes, which is involved in bactericidal function, inflammation, immunity, and hemostasis. In more highly evolved creatures, there are five types of white blood cells and platelets which respectively take care of specific functions, and platelets have long been considered to be an expert in thrombosis and hemostasis but nothing else. However, as described above, platelets are now increasingly considered to play as liaison interactive effects between different cell types and tissues.

Platelets are the second most abundant cell type in the circulation after red blood cells. Platelets contain three types of cytoplasmic granules, including α-granules, dense granules and lysosome, which contain a large number of autocrine and paracrine substances. Recent reports demonstrate that intra-granular proteins are differentially released upon different stimulation, suggesting that platelets can provide specific substances to specific tissues in appropriate conditions. In addition to its vast number in circulation, its small size and its ability to form microparticles which trespass various tissues, allows the detection of small breaches in any space as well as in the circulation. Microparticles are small fragments of membranes shed by mechanisms often involving metalloproteases. While leukocytes and endothelial cells can also produce microparticles, the majority (70 to 90 %) of microparticles in the circulation is considered to come from activated platelets. Platelet-derived microparticles express adhesive molecules and they may also contain proinfmmatory molecules such as IL-1β which may communicate proinflammatory signals to extravascular tissues. Furthermore, platelet-derived microparticles are also known to contain microRNA, which, upon release from microparticles, may be transferred to other cell types, where they modulate inflammation and immunity, as well as in developmental biology and angiogenesis. Thus, platelets and their microparticles can be figuratively described as the best drug-delivery system in living things [1].

## Discovery of CLEC-2

Rhodocytin, a snake venom obtained from the Malayan pit viper, *Calloselasma rhodostoma* activates platelets with a manner similar to collagen. Rhodocytin affinity chromatography and TOF-MASS spectrometry were utilized, and we identified a new class of platelet activation receptor, c-type lectin-like receptor 2 (CLEC-2) [2].

**Fig. 1:**
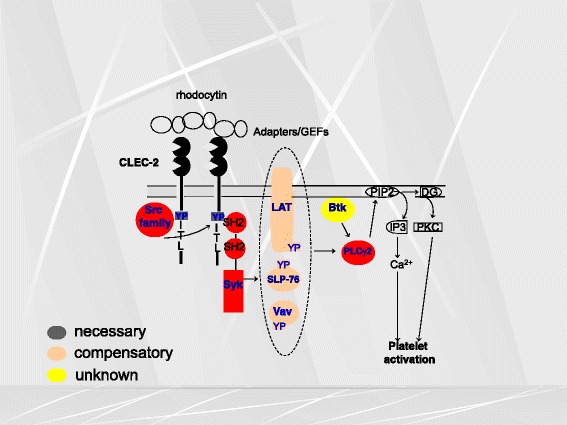
Signal transduction pathway mediated through CLEC-2. Overall, the signal transduction pathway is strikingly similar to that of GPVI, involving a number of signaling molecules related to tyrosine kinases. Upon association with its agonists, CLEC-2 assumes multimerization, and Syk and Src family kinases mediates tyrosine phosphorylation of its hemITAM, which is followed by downstream signals culminating in PLCγ2 activation. Signaling molecules required for full activation of platelets are marked in *red*, those which are partially required are marked in *orange*, and those which can be spared are marked in *yellow*

CLEC-2 belongs to the family of the non-classical C-type lectins. It contains one C-type lectin-like domain (CLTD) but lacking the consensus sequence for binding sugars and calcium. CLEC-2 assumes two forms, 32- and 40-kDaMW with varying degrees of glycosylation, in platelets. Its cytoplasmic tail contains a conserved YxxL sequence. The immunoreceptor tyrosine-based motif (ITAM) which plays an essential role of signal transduction in GPVI-related platelet activation has two tyrosine residues (tandem YxxL motif). In ITAM-related activation, both tyrosine residues undergo phosphorylation, and Syk with two SH2 domains which recognize phosphorylated tyrosine binds to this ITAM. In contrast, CLEC-2 has only a single cytoplasmic YxxL motif (hemITAM). However, we found that the intracellular signaling pathway elicited by CLEC-2-rhodocytin interaction is quite similar to that of GPVI (Fig. 1). Recent findings suggest that CLEC-2 exists as monomers and dimers at the resting state, and that when platelets are activated with CLEC-2 agonists, it assumes more profuse dimer formation or oligomerization, with resultant Syk interaction.

While CLEC-2 is expressed to a limited degree in liver sinusoidal endothelial cells, and liver Kupffer cells, CLEC-2 is abundantly and specifically expressed on platelets and megakaryocytes in humans . In mice, in addition to platelets, neutrophils and macrophages also express CLEC-2, and it appears to mediate phagocytosis and proinflammatory cytokine expression.

## Lymphangiogenesis

**Fig. 2:**
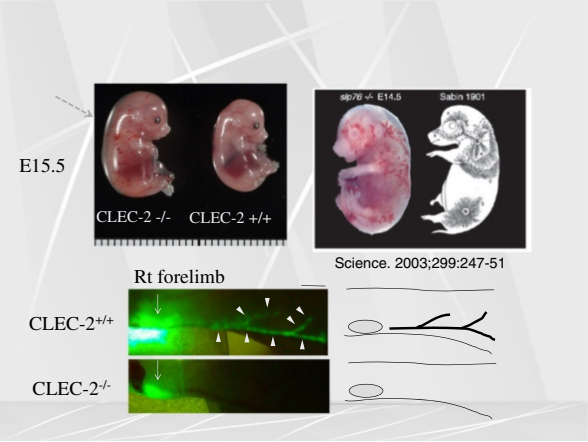
CLEC-2 knockout mice have abnormal lymphatic vessels. CLEC-2 knockout mice (*upper left inlet*) are edematous with dilated vessels, the pattern of which is quite similar to the distribution of lymphatic vessels of porcine fetus (*upper right inlet*), which was reported previously elsewhere. We found that the injected dye does not run into lymphatic vessels of the CLEC-knockout, suggesting for the presence of lymphatic vessel malformation (*lower inlets*)

Podoplanin was identified as an endogenous ligand for CLEC-2 [3]. Podoplanin is a sialo-glycoprotein, which is extensively O-glycosilated. It is highly expressed in renal podocytes, the name of which derives from, but it is also present in lung type I alveolar macrophages and lymphatic endothelial cells (LEC). In fact, it is often used as a marker for lymphatic vessels. It is also present on some types of tumor cells, and is responsible for tumor cell-induced platelet aggregation and tumor metastasis. A role for CLEC-2 in the developmental stage has been shown with CLEC-2 knockout mice with the phenotypes of blood-lymphatic vessel malseparation and edema [4, 5] (Fig. 2). Mice lacking signaling molecules such as Syk, PLCγ2, SLP-76, also manifest the similar phenotypes, and it is of interest that these signals are utilized by CLEC-2 for platelet activation. Knockout mice of podoplanin, the ligand for CLEC-2, also have blood lymphatic vessel malseparation and edema. One the other hand, mice lacking GPVI which also use the similar signaling pathway for platelet activation do not have this kind of phenotypes, suggesting that podoplanin-CLEC-2-interaction play an essential role. Since LEC lack these signaling molecules, we assumed that platelet activation, elicited by the binding between CLEC-2 on the platelet membrane and podoplanin on the surface of LEC podoplanin, is required for normal separation of blood/lymphatic vessels in the developmental stage. However, since neutrophils and dendritic cells in mice also express CLEC-2, and these signaling molecules are also present in those cells, we sought to confirm this hypothesis by producing platelet-specific CLEC-2 knockout mice.

**Fig. 3:**
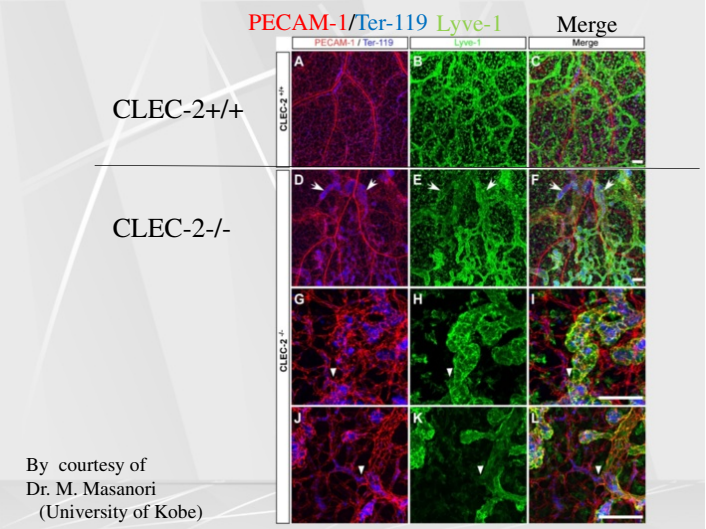
CLEC-2-knockout mice have dilated, torturous lymphatic vessels. While blood vessels and lymphatic vessels are distinctly separated in wild-type, they were intermingled with each other with CLEC-2-knockout mice, and the lymphatic vessels are dilated and torturous. PECAM-1 stains blood vessels, and Lyve-1 stains lymphatic endothelial cells. At the sites indicated by *arrowheads*, blood vessels and lymphatic vessels appear to be connected

In line with our expectation, platelet-specific CLEC-2 knockout mice manifest edema, lymphatic vessel dilatation, and the leakage of blood cells in lymphatic vessels [6] (Fig. 3). We have reached a conclusion that CLEC-2 expressed in platelets but not in other cells plays an important role in the normal separation of blood/lymphatic vessels. However, in contrast to pan-CLEC-2 deficient mice, the platelet-specific CLEC-2 mice, are not lethal at the stage of fetus or neonates, suggesting that CLEC-2 expressed in cells other than platelets are important somehow in maintaining life in the developmental stage.

**Fig. 4:**
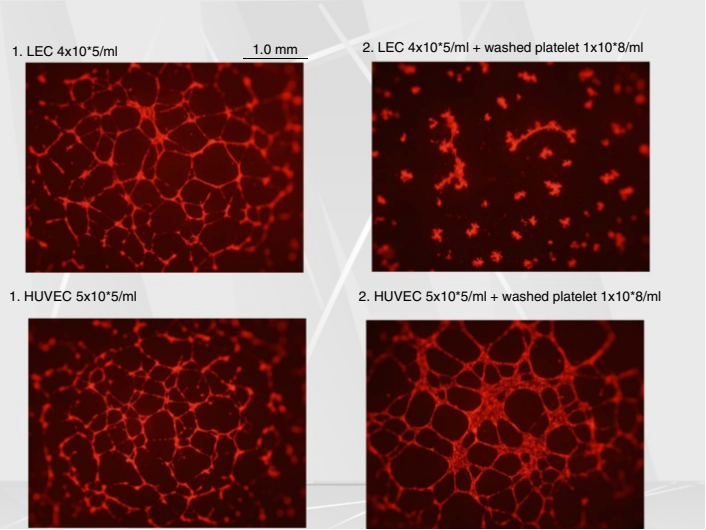
The supernatant of activated platelets inhibited tube formation of lymphatic endothelial cells (LEC) but not that of human umbilical venous endothelial cells (HUVEC). The *upper left inlet* shows the pattern of LEC tube formation without platelets, and the *upper right inlet* shows that LEC tube formation is disturbed in the presence of washed platelets which express CLEC-2. On the other hand, tube formation of HUVEC (*lower left inlet*) is not affected in the presence of platelets (*lower right inlet*)

There remains an interesting and important issue as to the mechanism by which the binding between podoplanin on the surface of LEC and CLEC-2 on the platelet surface regulates the lymphatic/blood vessel separation. Wild-type platelets with CLEC-2, but not CLEC-2 deficient platelets, inhibited LEC migration, proliferation and tube formation when co-incubated with LEC, and that granule contents released from activated platelets were responsible for this inhibitory effects. We found that cytokines belonging to the TGF-β superfamily play a role for this effect [6] (Fig. 4). Taken together, we suggest that the separation of lymphatic vessels from blood vessel during the developmental stage is regulated by cytokines released from platelets upon the interaction between CLEC-2 on the platelet membrane and podoplanin on lymphatic endothelial cells.

## Thrombosis and hemostasis

**Fig. 5:**
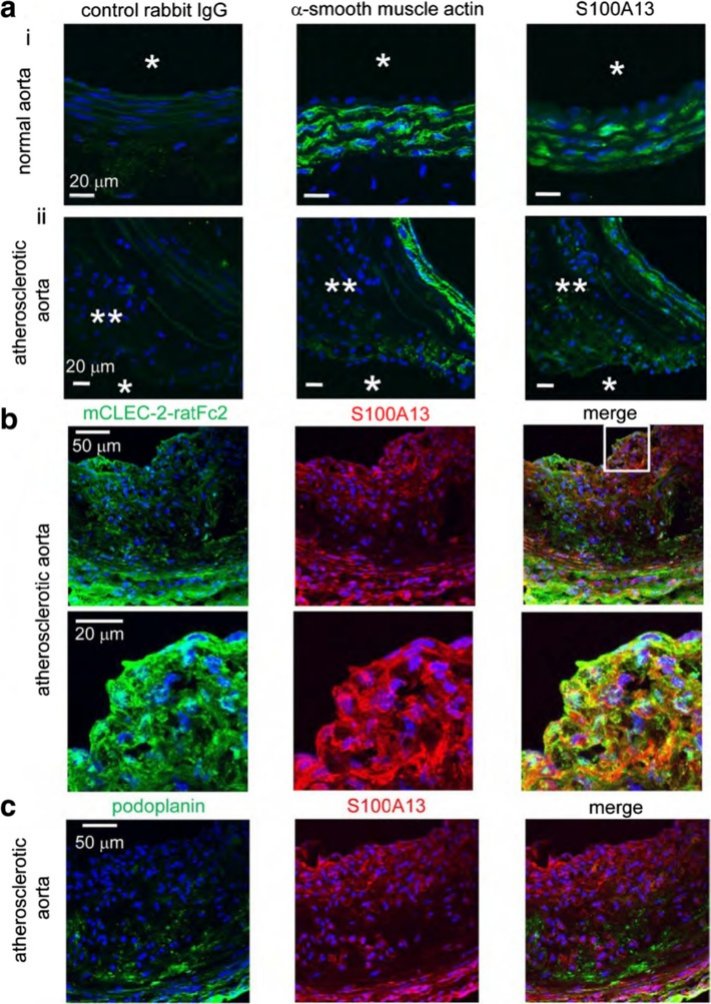
S100A13 is present in the normal aorta, and its distribution coincides with that of smooth muscle cells. In figures **a**, the distribution patterns of smooth muscle actin is similar to that of S100A12. Figures **b** show that CLEC-2 binding is abundantly present in atherosclerotic aorta, but less so in the normal aorta, and the binding of CLEC-2 colocalizes with that of S100A13. Podoplanin which is a ligand for CLEC-2 is expressed in the atherosclerotic tissues. However, the distribution of podoplanin is distinct from that of S100A13 (figures **c**), suggesting that CLEC-2 binding in the normal and atherosclerotic aorta is attributed to its binding to S100A13, but not to podoplanin

We generated chimeric mice whose blood cells were derived from the liver cells of CLEC-2-knockout fetus, in order to evaluate the role of CLEC-2 in thrombosis and hemostasis, since CLEC-2-knockout mice were lethal at the fetus/birth stage. Platelets taken from CLEC-2(−) chimeric mice had normal functions such as platelet adhesion and spreading. However, multilayer formation of platelets under the flow system or in vivo thrombus formation was significantly suppressed, in comparison to wild-type mice [5]. Stable thrombus formation under high shear stress requires the expression of CLEC-2 in platelets [7]. Although podoplanin may be focally detected in advanced atherosclerotic lesions [8], it is not expressed in normal subendothelial matrix, suggesting that there should be some ligands other than podoplanin for CLEC-2. We next searched for CLEC-2 ligands in normal vessel walls. Recombinant CLEC-2 bound to early atherosclerotic lesions and normal arterial walls, whose localization coincided with vascular smooth muscle cells (VSMCs). Flow cytometric study and immunocytochemistry revealed that recombinant CLEC-2, but not an anti-podoplanin antibody, bound to VSMCs, suggesting that VSMCs express certain CLEC-2 ligands other than podoplanin. The time to occlusion in a FeCl3-induced animal thrombosis model was significantly prolonged in CLEC-2 knockout mice. Since our FeCl3-induced injury model causes laceration in the internal elastic lamina, we assume that the interaction between CLEC-2 and its ligands in VSMCs exposed by laceration induces thrombus formation. Using protein arrays and Biacore analysis, we identified S100A13 as a CLEC-2 ligand in VSMCs [9]. S100A13 was released upon oxidative stress, and expressed in the luminal area of atherosclerotic lesions. Its staining pattern is distinct from podoplanin, and unlike podoplain which is only expressed in atherosclerotic lesions, but not in normal vessels, S100A13 is present in the normal vasculature, and its expression is enhanced in the atherosclerotic lesions (Fig. 5). However, it appears that there is as-yet unidentified CLEC-2 ligand in VSMCs which potently activates platelets, since S100A13 remains to be relatively weak in platelet activation.

Previously, CLEC-2 was considered to play only a minor role in hemostasis, because CLEC-2 knockout resulted in no or only a moderate increase in bleeding time. However, a recent report on severely defective hemostasis in GPVI and CLEC-2 double knockout mice suggests that GPVI and CLEC-2 compensate for each other, preventing severe blood loss, that they serve together to play a key role in hemostasis [10]. In the closely related context, it is of note that GPVI and CLEC-2 with the shared signal transduction pathways such as Syk, SLP-76, and PLCγ2 play a critical role in maintaining vascular integrity during development and inflammation [11].

## Role of platelets in liver regeneration

There is an increasing body evidence to suggest that platelets are involved in various stages of liver regeneration. They are not only involved in the early phase of liver generation, but platelet transfusion and thrombocytosis can enhance hepatocyte regeneration after liver injury. Recent studies have reported that platelets are recruited to the sinusoidal and Disse’s space and probably due to direct contact with certain cell types such as stellate cells and sinusoidal endothelial cells, they release bioactive compounds which stimulate hepatocyte proliferation [12]. However, up to date, the molecules on the platelet membranes which are involved in this process have not been identified.

We have recently found that hepatocyte proliferation is attenuated by clopidogrel, which inhibits platelet activation, and that liver regeneration is impaired with CLEC-2-knockout mice, suggesting that CLEC-2 on the platelet membrane is the key molecule which links with platelets and hepatocyte proliferation (manuscript being submitted).

## Megakaryopoiesis and thrombopoiesis

**Fig. 6:**
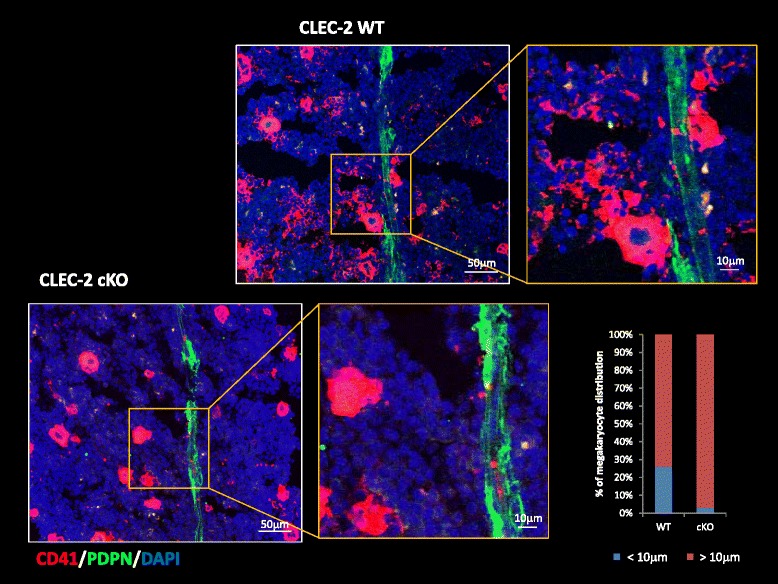
CD41^+^ clusters were formed adjacent to the podoplanin^+^ stromal cells in the BM CD41^+^ clusters which represent megakaryocytes were observed, lying close to the podoplanin^+^ stromal cells lining vasculature in the bone marrow. However, this phenomenon is not present with CLEC-2 knockout mice. The *right inlet* shows the quantitative distribution of megakaryocytes within 10 μm of vasculature in wild-type mice vs. CLEC-2 knockout

**Fig. 7:**
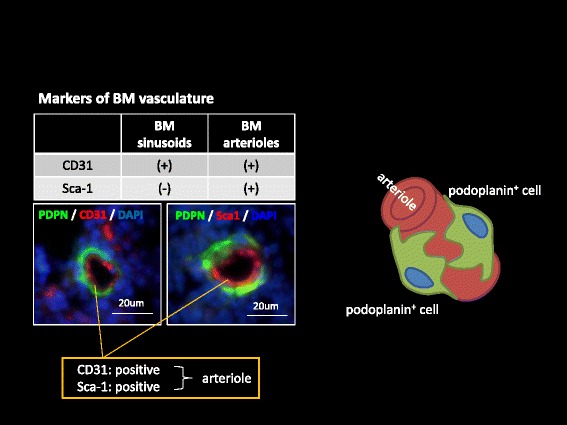
BM arteriolar stromal cells are podoplanin-positive. There are three type of vessels in the bone marrow, arterioles, sinusoids and lymphatic vessels. Only the bone marrow (BM) arteriolar stromal cells (CD31- and Sca-1-positive) are podoplanin-positive, and these cells are tentatively termed as BM fibroblastic reticular cell (FRC)-like cells. BM-FRC-like cells surround arterioles as illustrated in the *right inlet*

**Fig. 8:**
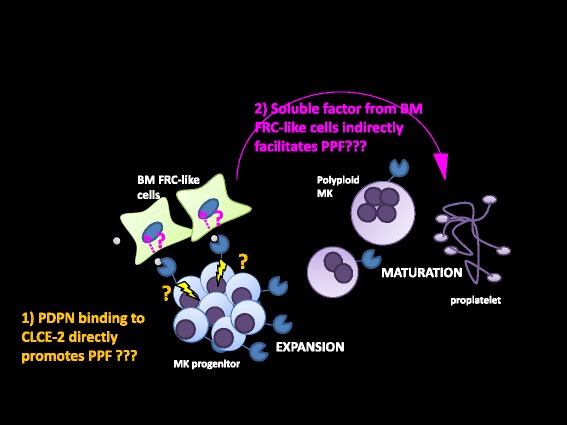
Hitherto, we have found that podoplain/CLEC-2 interaction induces megakaryocyte expansion. We then asked whether podoplain/CLEC-2 interaction acts on megakaryocytes to induce proplatelet formation, or on BM FCR-like cells which then contributes to proplatelet formation. Two hypotheses are depicted in Fig. 8

**Fig. 9:**
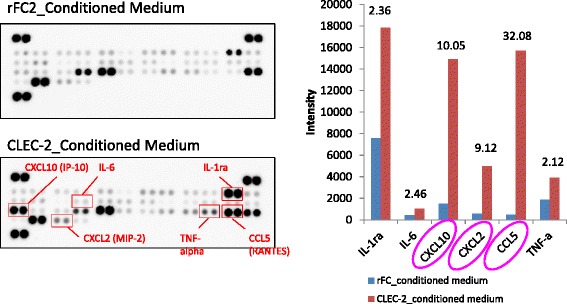
Proteome Profiler_Cytokine array. We found that some substances released from BM FCR-like cells upon interaction with CLEC-2-positive megakaryocytes serve to induce proplatelet formation. By the use of proteome profile cytokine array, three cytokines were identified, CXCL10, CXCL2, andCCL5. CCL5 was identified to be the most potent molecule released from BM FCR-like cells to induce proplatelet formation in megakaryocytes

**Fig. 10:**
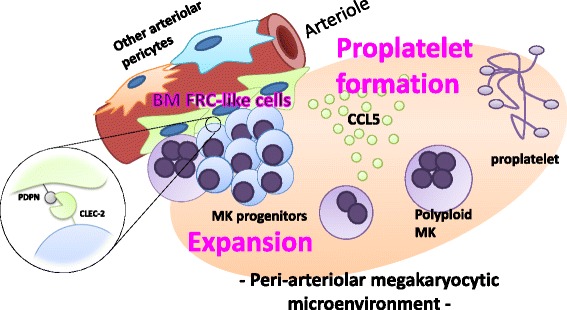
Microenvironment for megakaryopoiesis related to CLEC-2/podoplanin interaction. Our finding in this study suggest that a reciprocal interaction with between CLEC-2 on megakaryocytes and podoplanin on BM FRC-like cells contributes to the periarteriolar megakaryopoietic microenvironment in mouse BM

Megakaryopoiesis encompasses the sequential differentiation of hematopoietic stem cells into megakaryocytes, followed by thrombopoiesis in which maturation of megakaryocytes occurs with production of numerous platelets. CLEC-2 is expressed on platelets and megakaryocytes, and thrombocytopenia occurs with deletion of platelet/megakaryocyte CLEC-2 in mice. Megakaryopoiesis is promoted through the CLEC-2/podoplanin interaction in the vicinity of vessels, and we found that this occurs in the vicinity of arterioles, not sinusoids or lymphatic vessels (Fig. 6). We found that podoplanin-expressing stromal cells exit adjacent to BM arterioles, and tentatively termed these cells as BM fibroblastic reticular cell (FRC)-like cells (Fig. 7). There was a significant decrease in the number of immature megakaryocytes in platelet/megakaryocyte-specific CLEC-2 conditional knockout (cKO) mice. CLEC-2 WT megakaryocyte expansion was enhanced *in vitro* by the addition of recombinant podoplanin, but this did not occur with cKO megakaryocytes. Furthermore, megakaryocyte colonies appeared to nest adjacent to periarteriolar BM FRC-like cells in the BM. Co-culture of megakaryocytes with BM FRC-like cells maintained megakaryocyte expansion, which appeared to be dependent upon the CLEC-2/podoplanin interaction. We then asked whether podoplain/CLEC-2 interaction acts on megakaryocytes to induce proplatelet formation, or on BM FCR-like cells which then contributes to proplatelet formation, in addition to the megakaryocyte expansion (Fig. 8). We found that the CLEC-2/podoplanin interaction induces BM FRC-like cells to secrete CCL5 to facilitate proplatelet formation (Fig. 9). These observations suggest that a reciprocal interaction between CLEC-2 on megakaryocytes and podoplanin on BM FRC-like cells contributes to the megakaryopoietic microenvironment in the periarteriolar space in mouse BM [13] (Fig. 10).

## Conclusions

There is an increasing body of evidence to suggest that platelets participate in various patho-physiological processes beyond those related to thrombosis and hemostasis, and that CLEC-2 on the platelet membrane by interacting with its ligands, podoplanin and others, contributes to a number of these processes.

## Abbreviations

CLEC-2: C-type lectin-like receptor 2
LEC: Lymphatic endothelial cell
VSMC: Vascular smooth muscle cell
HUVEC: Human umbilical vein-derived endothelial cell
TLR: Toll-like receptor
BM: Bone marrow
NET: Neutrophil extracellular trap
FRC: Fibroblastic reticular cell
